# Effects of beak-treatment styles on beak morphology and production performance of layer chicks aged 0–8 weeks

**DOI:** 10.3389/fvets.2025.1546993

**Published:** 2025-03-13

**Authors:** Lin Duan, Xiao-Yu Zhao, Er-Ying Hao, Chuan-Wen Wang, De-He Wang, Hui Chen, Zhong-Qiang Wang, Li-Jun Xu, Ke-Qian Di

**Affiliations:** ^1^College of Animal Science and Technology, Hebei Agricultural University, Baoding, China; ^2^Baoding Xingrui Agriculture and Animal Husbandry Development Co., Ltd., Xushui, China; ^3^Beijing Egg Industry Association, Beijing, China; ^4^Baoding Livestock Science and Technology, Baoding, China; ^5^School of Basic Medical Sciences, Hebei University, Baoding, China

**Keywords:** layer chick, infrared, laser, beak shape, growth and development

## Abstract

**Introduction:**

Double-infrared (IR-D) and laser energy sources (LAS) are two types of newly beak treatment equipment. Although the IR-D and LAS techniques have been developed by changing the number and type of energy sources, their effects on layer chicks are yet to be systematically studied.

**Methods:**

In this study, we placed 4,416 layer chicks into groups subjected to single-infrared energy sources (IR-S), IR-D, and LAS beak treatment on hatching day and a sham untreated control (CON) group; the beak sloughing, growth and histomorphology, body weight (BW), feed intake (FI), and other performance indicators were measured.

**Results:**

Results showed that the beak length at 4 to 8wk in LAS group was significantly higher than that in IR-S and IR-D groups (*p* < 0.05), and showed a better symmetry. The beak bone mineral density (BMD) and the beak bone mineral content (BMC) at 3–4 weeks was the lowest in the CON group. The BW at 1–4 weeks and the FI at 1 and 3 weeks in the CON group were significantly higher than those in the IR-S and IR-D groups (*p* < 0.05), whereas there was no significant difference between the BW and FI of the CON and LAS groups from 1 to 8 weeks. The liver weight percentage from 1 to 2 weeks and the bursa Fabricius weight percentage at week 8 in the CON group were significantly higher than those in the experimental groups (*p* < 0.05).

**Discussion:**

This study concluded that the LAS group had certain advantages in beak length, symmetry, BW, and FI and provided a reference for evaluating the effects of beak treatment methods on layer chicks.

## Introduction

Beak trimming, is a strategy that is used to curb cannibalistic tendencies in commercial hens and was introduced in 1937 (1). The purpose of beak trimming is to reduce or inhibit aggression and cannibalism in hens. Studies have shown that hot blade beak trimming could decreased cannibalism-related mortality by 35–45% (2) and reduced daily feed wastage per bird by 3–10 g (3). The hot blade beak trimming, which cuts and cauterizes the beak at approximately 750°C (4), is effective but has been criticized because of the pain and distress inflicted on the birds, supported by evidence from morphological, neurophysiological, and behavioral studies (5–7). Thus, an exploration of alternative methods that ensures both bird welfare and operational efficiency is necessary.

In 2005, the first report on infrared (IR) beak treatment emerged (8). This technique employs a single-IR energy source, with a wavelength of 5–6 μm, to penetrate the beak’s rhamphotheca layer via a 360-degree reflector, damaging the underlying tissue layers (9). The IR-S method offers advantages over hot blade treatment, including no open wounds and reduced stress from treatment and handling (9). However, some studies have indicated that IR-S treatment may lead to decreased feed intake (FI) and body weight (BW) in chicks (3, 10, 11).

Advancements in technology have introduced double-IR (IR-D) and laser (LAS) sources, which are anticipated to enhance future treatments. The IR-D method operates similarly to IR-S, utilizing two independent IR sources to target and treat the top and bottom beak tissues (10). The LAS method employs a carbon dioxide (CO2) laser with a wavelength of 10.6 μm, which is focused through a lens for precise beak treatment. CO2 lasers are valued for their precision in cutting and good hemostasis, particularly in ophthalmic surgery (12–16). Although the application of this technology in poultry beak trimming has been explored, there are limited reports on its effectiveness.

The aim of this study was to systematically assess the impact of various beak-treatment methods, including IR-S, IR-D, and LAS, on beak integrity, morphology, and growth performance indicators in commercial layer chicks from 0 to 8 weeks of age. The findings inform the refinement of beak-treatment technologies.

## Materials and methods

### Layer chicks

All experiments in this study were conducted in accordance with the Guidelines for the Care and Use of Laboratory Animals, prepared by the Institutional Animal Care and Use Committee of Hebei Agricultural University, Baoding, China.

In total, 4,416 female commercial hens (Lankao Xiaoming Poultry Industry Co., Ltd., Henan, China) were selected and divided into four groups averagely (1,104 layer chicks per group): IR-S, IR-D, LAS, and sham untreated control (CON). A 42 W IR-S Poultry Service Processor (PSP, Nova-Tech Engineering LLC, MN, United States), a 60 W IR-D beak-treatment machine (KJ03-0090, Dongguan Kaijie Automation Technology Co., Ltd., Dongguan, China), and a 30 W LAS Poultry Service Processor (Poulcare-Tech Co., Ltd., Shenzhen, China) were used by trained operators for beak treatment immediately after hatching. The top and bottom beaks of chicks from each group were treated using the corresponding beak-treatment equipment, with approximately half of the top beak and one third of the bottom beak being treated, respectively. The CON group served as a sham, untreated control. Chicks in the CON group were placed into the IR-S beak treatment equipment, but their beaks remained untreated. Next, the chicks from the four groups (*n* = 46 per replicates) were raised in a fully enclosed house, with an automatic H type layer cage system (9CLD-3180, Retech System LLC, Galeata, Italy); the size of an individual cage was 80.5 × 87.5 × 39 cm. The stocking densities were as follows: 46 chicks per cage during 1st week, 30 or 31 chicks per cage during 3–4 weeks, and 23 chicks per cage during 4–8 weeks. The temperature was set at 35–36°C for the initial brooding period (3 d) and then reduced by approximately 2°C per week to reach a final temperature of 19°C at 8 weeks of age. Humidifiers were used to maintain a relative humidity range of 40–75%. The photoperiod was 24 L:0 D (40 lux) for the first 3 d. At 4–7 d of age, the photoperiod was 22 L: 2 D (25–40 lux). Starting at 8 d of age, the photoperiod and light intensity were reduced by 2 h and 5lux, respectively, per week to reach a final photoperiod of 11 L:13 D (5 lux). Food and water intake and other feeding management conditions were consistent with the recommendations of the Hy-Line Brown Feeding Management Manual. The experimental period was 8 weeks, and the contents and methods of the measurement indices were as follows.

### Beak sloughing and morphology

**Figure 1:**
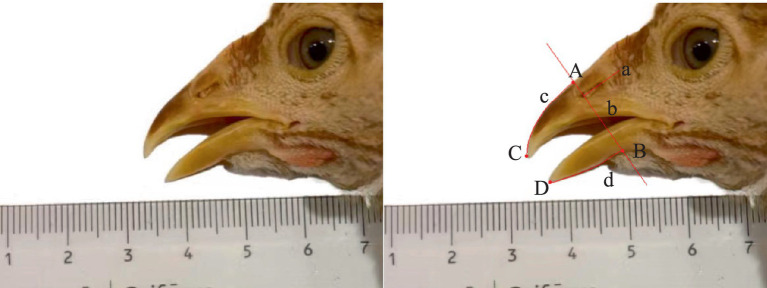
Illustration of beak length analysis by Image J software (Left: a common ruler scale, right: illustration of top and bottom beak length). A, start point of top beak length; B, start point of bottom beak length; C, end point of top length; D, end point of bottom length; c, top beak length; d, bottom beak length.

From 7 to 28 d of age, 50 chicks in each of the four groups, i.e., a total of 200 chicks, were randomly captured every day to observe and record whether their top and bottom beaks sloughed. On the 4th day of their 1st, 2nd, 3rd, 4th, 6th, and 8th weeks, 50 chicks were selected from each group for beak images. Digital images of the subjects’ beaks were captured using a Nikon D3400 kit camera (Nikon Europe BV, Amstelveen, Netherlands). These images were subsequently imported into the Image J software (National Institutes of Health (NIH), Bethesda, MD, United States) for precise calibration to ascertain the dimensions of the top and bottom beak segments. The specific methods employed for measurement are delineated in Figure 1. The methodology was refined in accordance with the protocols established by Cheng et al. (11). First, a straight Line a was drawn through the long axis of the nares. Second, a straight Line b, perpendicular to Line a, was drawn through the tip of the nares. Straight Line b intersected with the outer edges of the top and bottom beaks and Points A and B, which were used as the starting points for measuring the top and bottom beak lengths, respectively. Third, the farthest ends of the top and bottom beaks were set as Points C and D, and a smooth curve was used to connect points A and C and points B and D along the outer edge of the beak to form curves c and d, where curves c and d are the lengths of the top and bottom beaks, respectively. Differences in the top and bottom beak lengths and symmetry (the length of the top beak extending out of the bottom) among the four groups were compared.

### Beak tissue

According to the regularity of beak sloughing-off time in the IR-S group (17) five chicks in each group were randomly selected and euthanized by manual cervical dislocation after beak treatment on days 1 (2 d of age), 6 (7 d of age), and 16 (peak of beak sloughing; 17 d of age), respectively. The top and bottom beaks were removed from the posterior position of the top nasal bone. The beaks were then immersed in 10% neutral formalin (F980839; Macklin Biochemical Technology Co., Ltd., Shanghai, China) at a volume ratio of 1:5 and placed at room temperature for 1 week to fix the morphology. Subsequently, the fixed beak tissue was decalcified in EDTA standard solution (PST008, Phygene Biotechnology LLC, Beijing, China) at a volume ratio of 1:25 for 1 week. The decalcified beak tissue was embedded in paraffin wax, sectioned at 5 μm (Shandon Finesse, Thermo Electron Company, Inc., Pennsylvania, United States), and stained with hematoxylin and eosin (HE; SelecTech Hematoxylin 560 and SelecTech Alcoholic Eosin Y515, Leica Biosystems, Winnipeg, MB, Canada).

**Figure 2:**
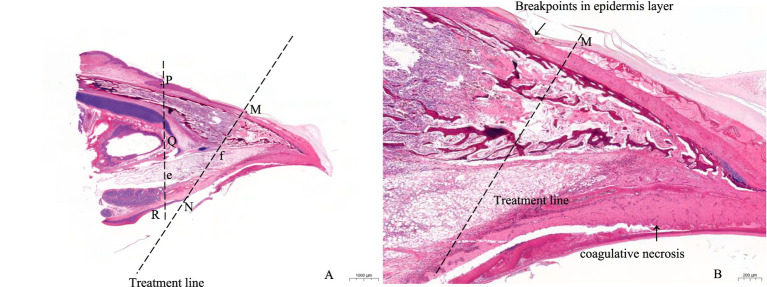
Schematic illustration of a histological section of the top beak after treatment with a single infrared energy source. P, anterior end of the outer nostril; Q, anterior part of intranasal cavity; R, extension point of point P and Q; M, start point of beak treatment; N, end point of beak treatment; e, reference line; f, beak treatment line. A, B: Histological section of the top beak of 2d of age layer chick after infrared beak treatment at 10×, 40× magnification.

Beak tissue indicators, namely epidermal layer thickness and length percentage, necrotic beak area, and the number of Herbst corpuscles (nerve endings) (18) in each group, were observed and recorded using an optical microscope (M330, Aosvi Optical Instruments Co., Ltd., Guangzhou, China) under a magnification of 10× or 40×, which were processed using ImageJ v1.52 (National Institutes of Health) analysis software. The detailed measurement methods of beak tissue are shown in Figure 2. Points P (anterior end of the outer nostril) and Q (anterior part of intranasal cavity) were connected to extend to Point R. The distance from Points P and R to the top and bottom beak tip was the whole epidermis length. The area from Line e to the beak tip was the entire beak area. After the IR beak treatment, a cracked epidermal layer (Point M) and a clear boundary between the left and right sides of the treatment line were observed. The area from Line f to the beak tip represented the necrotic beak area. Therefore, the percentage of epidermal layer length was calculated as the ratio of anterior nasal untrimmed epidermal layer length to the entire beak length of the outer margin. In addition, we randomly selected three locations in the epidermal layer to measure the thickness, and the average of these three values was the thickness of the beak epidermal layer.

On the third day of the 2nd, 3rd, 4th, and 8th weeks of age, five chicks from each group were selected and humanely euthanized by manual cervical dislocation. The top and bottom beaks of each chick were removed to measure the bone mineral density (BMD) and bone mineral content (BMC) using Dual-energy X-ray Absorptiometry for small animals (InSiGHTVETDXA, Baitai Technology Co., Ltd., Guangdong, China).

### Growth performance and organ weight percentages

When the chicks were 1, 2, 3, 4, 6 and 8 weeks of age, six large cages (six duplicates) per group were maintained; all 24 cages were on the same floor, and were selected for each group to record the FI. The FI from the 2nd to the 5th at 1 week of age was recorded and calculated on a weekly basis. On the 2nd and 5th day of each week, 50 chicks from each group were randomly selected and weighed BW. The average weight for these 2 d was reported as the weekly weight. The BW and the weights of the heart, liver, spleen, bursa of Fabricius, and adrenal gland were measured using an electronic balance (JA1003, 0.001 g, Xiuilab Instrument Co., Ltd., Shanghai, China). Organ weight percentage was calculated as the ratio of the weight of each organ to its BW.

### Statistical analysis

Quality control was carried out for each indicator to reduce random errors. The abnormal values of beak length and body weight were removed using the Z-score method, and the abnormal values of beak morphology and percentage of organ weight were removed using the IQR method. The mean differences among the four groups of the above indicators were analyzed using one-way ANOVA and Duncan’s multiple comparison in IBM SPSS Statistics 27 (IBM Corp., Armonk, NY, United States). The beak symmetry difference was compared with the absolute value of the length of the top beak extending from the bottom beak. A χ^2^ independence test was used to determine the differences in beak sloughing cumulative proportions at different times; the level of significance difference was set at 0.05.

## Results and discussion

### Beak sloughing

**Figure 3:**
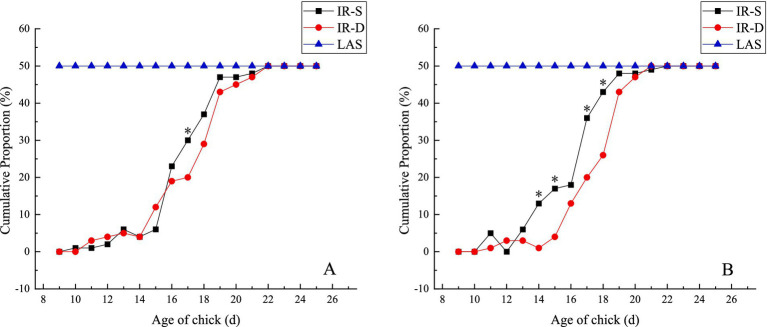
Effect of beak treatment on beak sloughing **(A)** top beak, **(B)** bottom beak. IR-S, single-infrared energy source treatment; IR-D, double-infrared energy source treatment; LAS, laser energy source treatment. * between IR-S and IR-D groups at the same age mean without a common superscript differ (*P*<0.05).

The cumulative proportion regularity of the top and bottom beak sloughing over time in the experimental groups is shown in Figure 3. With increasing age, beak sloughing did not occur in the CON group but immediately in the LAS group after laser treatment. Top and bottom beak sloughing began in the IR-S and IR-D groups from 9 to 11 d of age, and all beak sloughing was complete in both groups at 22 d of age. The cumulative sloughing proportion of the top beak at 17 d of age in the IR-S group was significantly higher than in the IR-D group. In the IR-S group, the cumulative sloughing proportion of the bottom beak at 14–15 and 17–18 d of age was significantly high (*p* < 0.05); and the cumulative sloughing proportions of the top beak at 16–22d and the bottom beak at 13–22d were higher than those observed of in the IR-D group (Figure 3B). Generally, the chick age at the top and bottom beak sloughs in the IR-S group was earlier than that of the IR-D group, which might have been due to the actual power of the equipment. The peak time of top beak sloughing in the IR-S and IR-D groups was mainly concentrated at 15–19 d of age, and the peak time of bottom beak sloughing in the IR-D group was consistent with the top beak sloughing in the IR-S and IR-D groups, which was also concentrated at 15–19 d of age. This is consistent with the results of IR-S beak sloughing by Struthers et al. (17). In the IR-S group, the peak time of bottom beak sloughing was mainly concentrated at 13–19 d of age, and the period was prolonged. The main components of the top and bottom beaks are bone salts of collagen-bonded layered hydroxyapatite crystals, organic matter, and small amounts of water (19). From a phenotypic point of view, the thickness of the top beak at the beak-treatment position was more than that of the bottom beak (20). It is possible that the thinner bottom beak and relatively higher equipment power caused the change in the peak period of bottom beak sloughing in the IR-S group.

### Beak length and symmetry

**Figure 4:**
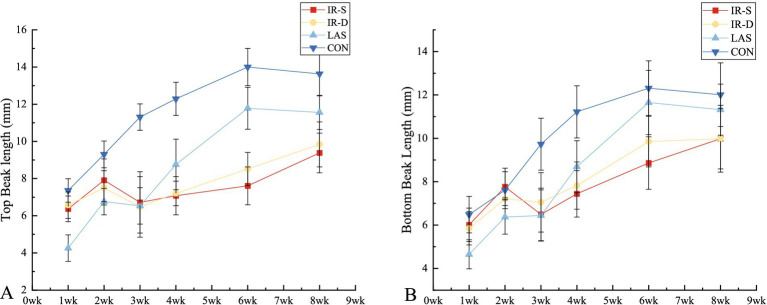
Effect of beak treatment on beak length **(A)** top beak, **(B)** bottom beak. IR-S, single-infrared energy source treatment; IR-D, double-infrared energy source treatment; LAS, laser energy source treatment; CON, sham untreated control.

The growth patterns of the top and bottom beaks in the different groups are shown in Table 1. The top and bottom beaks in the CON group were significantly longer than in the IR-S, IR-D, and LAS groups at 1–8 weeks (*p* < 0.05). These results suggest that different beak-treatment methods inhibit early beak regrowth (11). The top and bottom beak lengths of the IR-S and IR-D groups at 1–2 weeks of age were significantly higher than those of the LAS group because the beak sloughed off immediately after LAS beak trimming. The top and bottom beak lengths of the IR-S and IR-D groups from 4 to 8 weeks of age were significantly shorter than those of the LAS group (*p* < 0.05), indicating that the LAS group promoted beak growth compared with the IR-S and IR-D groups. Also shown in Figure 4, according to the slope of beak growth, the LAS group’s beaks grew faster than IR-S and IR-D group at 1–6 weeks of age. This growth rate is probably due to the following: during the acute phase of healing, there is less collagen damage and less heat necrosis, and the regeneration of epithelial and dermal fiber tissues is more robust (21, 22); thus, the LAS treatment healed faster and did not affect the normal growth of beak tissue. Furthermore, CO2 lasers are often used in the medical field because of their accuracy, efficiency, and safety, and previous studies have shown that they can promote the growth of skin collagen fibers (22–24). In the IR-D group, the top beak length at 6 to 8 weeks of age was significantly longer than that in the IR-S group, and the bottom beak length at 3, 6 weeks of age was significantly longer than in the IR-S group (*p* < 0.05). These results indicate that the IR-D group promoted beak growth compared with the IR-S group.

**Table 1:** Effect of beak treatment on top and bottom beak length in layer chicks from 1 to 8wk of age.

Beak length (mm)	Group	Items						P
		1wk	2wk	3wk	4wk	6wk	8wk	
Top	IR-S	6.37 ± 0.69^b^	7.91 ± 1.15^b^	6.72 ± 1.65^b^	7.08 ± 1.03^c^	7.61 ± 1.02^d^	9.38 ± 1.07^d^	<0.001
	IR-D	6.57 ± 0.71^b^	7.50 ± 0.92^b^	6.48 ± 1.63^b^	7.20 ± 0.66^c^	8.52 ± 0.89^c^	9.84 ± 1.21^c^	<0.001
	LAS	4.26 ± 0.71^c^	6.76 ± 0.71^c^	6.53 ± 0.98^b^	8.76 ± 1.36^b^	11.78 ± 1.13^b^	11.56 ± 0.92^b^	<0.001
	CON	7.36 ± 0.63^a^	9.30 ± 0.72^a^	11.31 ± 0.71^a^	12.29 ± 0.89^a^	14.00 ± 1.00^a^	13.63 ± 1.18^a^	<0.001
Bottom	IR-S	6.01 ± 0.77^b^	7.76 ± 0.86^a^	6.49 ± 1.21^c^	7.44 ± 1.07^c^	8.86 ± 1.21^d^	9.98 ± 1.39^c^	<0.001
	IR-D	5.85 ± 0.77^b^	7.21 ± 1.04^b^	7.05 ± 1.37^b^	7.82 ± 1.09^c^	9.85 ± 1.17^c^	9.98 ± 1.54^c^	<0.001
	LAS	4.65 ± 0.67^c^	6.37 ± 0.79^c^	6.44 ± 1.18^c^	8.69 ± 1.20^b^	11.65 ± 1.48^b^	11.32 ± 1.18^b^	<0.001
	CON	6.48 ± 0.84^a^	7.61 ± 0.85^ab^	9.73 ± 1.20^a^	11.22 ± 1.20^a^	12.31 ± 1.26^a^	12.01 ± 1.47^a^	<0.001

IR-S, single-infrared energy source treatment; IR-D, double-infrared energy source treatment; LAS, laser energy source treatment; CON, sham untreated control. ^a,b,c,d^ Among 4 groups means within a main effect with different superscripts are significantly different (*p* < 0.05). All values are mean ± standard deviation.

**Figure 5:**
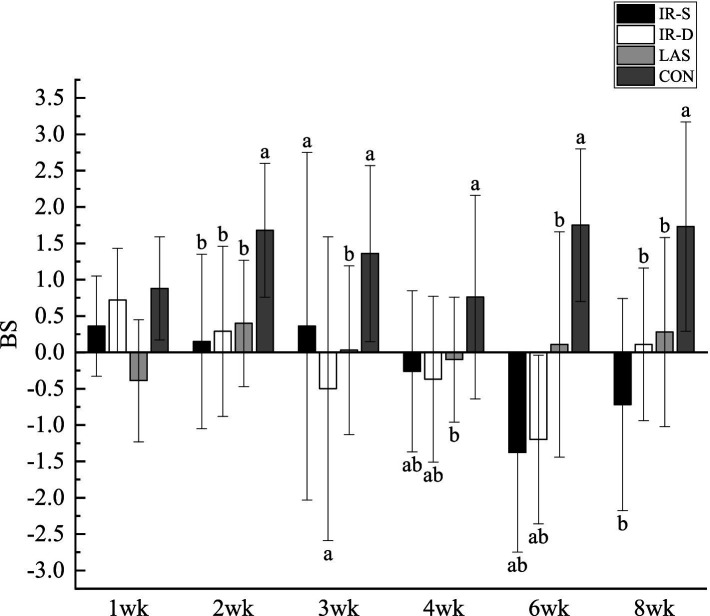
Effect of beak treatment on beak symmetry in layer chicks from 1 to 8wk. IR-S, single-infrared energy source treatment; IR-D, double-infrared energy source treatment; LAS, laser energy source treatment; CON, sham untreated control; BS, beak symmetry means the length of the top extending beyond the bottom. ^a,b^ Among 4 groups at the same age mean without a common superscript differ (*P*<0.05).

The results of beak symmetry (the length of the top beak extending beyond the bottom) are shown in Figure 5. From 2 to 8 weeks of age, the difference in length between the top and bottom beaks in the CON group was maintained at approximately 1.5 mm, and the degree of symmetry was significantly lower than that in the other three experimental groups (*p* < 0.05). In its natural state, the top beak exists in curvature; therefore, the top beak of the CON group was longer than the bottom beak (25). From 1 to 8 weeks of age, the beak symmetry in the LAS group was maintained within 0.5 mm, indicating good symmetry (26). The top beaks of the IR-S and IR-D groups were longer than the bottom beaks at 1–2 weeks of age and shorter than the bottom beaks at 4–8 weeks, and the beak symmetry of the IR-S and IR-D group at 3 weeks of age was significantly lower than that of the LAS group. Beak symmetry tended to fluctuate, consistent with the results of McKeegan et al. (27). This may have been caused by the regularity of beak sloughing and the periodicity of the top and bottom beak growth rates.

### Beak tissue

The effects of chicken beak treatment on BMD and BMC are shown in Table 2. From 2 to 8 weeks of age, the top and bottom beak BMC gradually increased. The pattern of bone development is similar to that of other body parts in chicks (28). The BMD and BMC of the top and bottom beaks at 3–4 weeks in the CON group had a significantly decreased (*p* < 0.05). However, there were no significant differences in the BMD and BMC in the top and bottom beaks between the control and experimental groups at the 8th week. These results suggest that beak treatment improves beak bone quality within a certain period and that these effects may be compensatory. As the beak contains approximately 44.0% defatted crude ash (29), bone mineral loss may reduce the BMD (30). Therefore, the BMD and BMC trends were similar. The 3-week top beak BMD was significantly higher in the LAS group than in the IR-S group, and the 2-week top beak BMC was significantly higher in the LAS group than in the IR-S and IR-D groups (*p* < 0.05). The 4-week top beak BMD and BMC and bottom beak BMD and BMC in the LAS group were lower than those in the IR-S and IR-D groups. This trend was reversed from 3 to 4 weeks of age, indicating that beak sloughing plays an important role in bone quality. From 2 to 8 weeks of age, there was little difference in the top and bottom beak BMD and BMC indicators between the IR-S and IR-D groups. The changes in beak quality among different groups corresponded to the changes in beak length shown in Table 1, and the relationship between beak quality and beak length was inverse from 2 to 4 weeks of age.

**Table 2:** Effect of beak treatment on BMD and BMC of layer chicks from 2 to 8wk of age.

Beak	Indictors	Age (weeks)	Group				P
			IR-S	IR-D	LAS	CON	
Top	BMD (×10^−3^ g/cm^2^)	2	17.5 ± 5.12	14.7 ± 4.11	16.8 ± 2.50	14.0 ± 1.83	0.655
		3	21.2 ± 6.62^bc^	26.7 ± 7.42^ab^	29.2 ± 3.11^a^	17.4 ± 3.36^c^	0.015
		4	36.8 ± 3.63^a^	34.2 ± 2.77^ab^	29.0 ± 4.97^b^	20.0 ± 5.29^c^	<0.01
		8	48.2 ± 4.97	50.0 ± 6.40	47.7 ± 6.32	44.7 ± 2.97	0.798
	BMC (×10^−3^ g)	2	0.8 ± 0.50^b^	1.3 ± 0.50^b^	2.5 ± 0.58^a^	1.25 ± 0.50^b^	0.003
		3	5.2 ± 0.75^a^	4.8 ± 1.94^a^	5.4 ± 1.14^a^	1.6 ± 1.52^b^	0.001
		4	7.6 ± 1.82^a^	8.2 ± 1.92^a^	4.8 ± 2.06^b^	2.8 ± 2.17^b^	0.002
		8	19.0 ± 1.00^b^	29.7 ± 4.04^a^	21.3 ± 5.06^b^	18.7 ± 2.08^b^	0.054
Bottom	BMD (×10^−3^ g/cm^2^)	2	25.0 ± 1.41^b^	22.8 ± 0.96^b^	25.0 ± 0.81^b^	28.3 ± 2.06^a^	0.001
		3	36.3 ± 3.20^a^	35.0 ± 1.90^ab^	33.8 ± 3.11^ab^	31.8 ± 4.26^b^	0.114
		4	41.6 ± 3.85^a^	42.2 ± 5.12^a^	35.5 ± 2.08^b^	35.2 ± 3.96^b^	0.036
		8	59.3 ± 5.13	57.0 ± 4.08	52.0 ± 6.93	56.6 ± 6.80	0.544
	BMC (×10^−3^ g)	2	2.8 ± 0.96	2.0 ± 1.00	2.0 ± 0.81	1.5 ± 1.00	0.315
		3	8.5 ± 1.87^a^	7.2 ± 2.40^a^	3.8 ± 1.79^b^	2.0 ± 1.10^b^	<0.001
		4	8.4 ± 2.30^a^	8.0 ± 2.92^ab^	5.8 ± 0.96^ab^	4.8 ± 2.17^b^	0.084
		8	22.7 ± 6.66	23.3 ± 6.51	18.3 ± 4.04	20.0 ± 6.56	0.850

IR-S, single-infrared energy source treatment; IR-D, double-infrared energy source treatment; LAS, laser energy source treatment; CON, sham untreated control. BMD, bone mineral density; BMC, bone mineral content. ^a,b,c^ Among 4 groups means within a main effect with different superscripts are significantly different (*P* < 0.05). All values are mean ± standard deviation.

**Figure 6:**
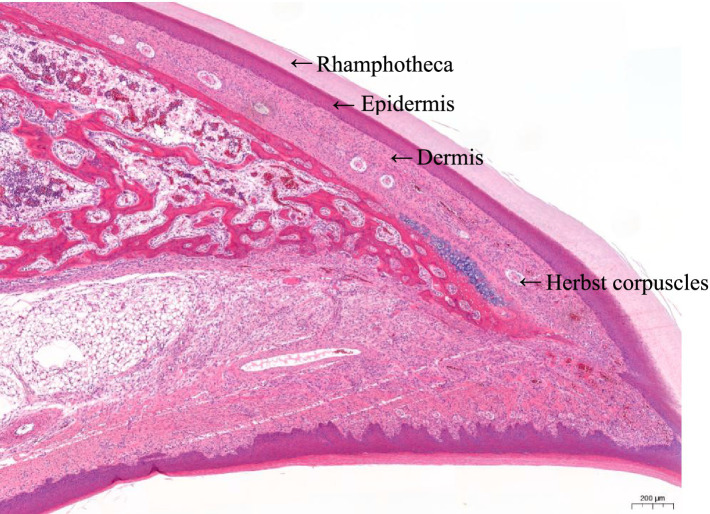
Schematic illustration of the top beak tissue sections of a chick from the control group. Beak tissue of three layers are indicated: rhamphotheca, epidermis, and dermis. Nerve endings: Herbst corpuscles are visible within the dermis layer.

**Figure 7:**
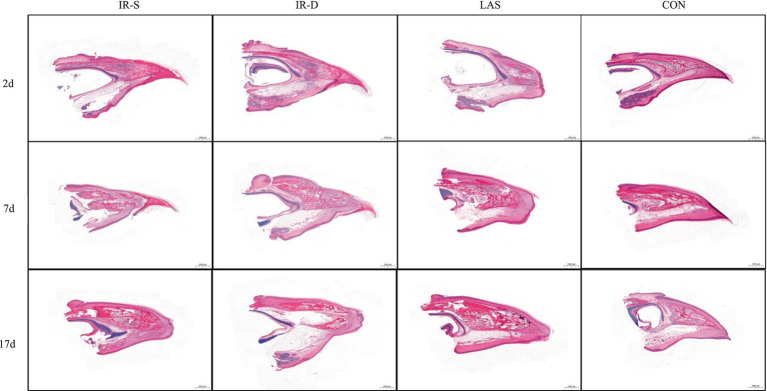
Process of beak healing, growth and sloughing of different beak-treatment styles during 2 to 17 d of layer chick age. IR-S, single-infrared energy source treatment; IR-D, double-infrared energy source treatment; LAS, laser energy source treatment; CON, sham untreated control.

The beak tissue structure is mainly divided into the rhamphotheca, epidermis, and dermis layers (18), and the dermis layers contain a large number of blood and nerve endings (Herbst corpuscles; Figure 6) (31). The effect of infrared beak treatment on beak tissue is shown in Figure 2. There was a clear boundary between the anterior and posterior ends of the beak treated with infrared radiation and coagulative necrosis of the beak epithelium (32). The necrotic beak tissue may exhibit cellular infiltration, edema and inflammatory infiltration (Figure 7). The results of beak tissue structure post beak treatment, including the thickness and length percent of the epidermal layer and the number of Herbst corpuscles, analyzed quantitatively, are shown in Table 3. At 17 d of age, the epidermis layer thickness of the CON group was significantly higher than that of the IR-S, IR-D, and LAS groups, and the epidermal thickness of the IR-S group was significantly lower than that of the IR-D and LAS groups (*p* < 0.05). These three methods of beak treatment inhibited the growth of the beak tissue at the cellular level, which is consistent with the results of Henderson et al. (33) and Struthers et al. (34). Considering the epidermis thickness and length, the epidermal length percentage of the LAS group was lower than that of the IR-D and LAS groups; the IR-S equipment had a more obvious inhibitory effect on the epidermal thickness. From 2 to 7 d of age, the percentage of epidermal length in the CON group was significantly higher than that in the other three experimental groups (*p* < 0.05); in the LAS group, the percentage of epidermal length was significantly higher, and the percentage of necrotic beak tip area was significantly lower, than those in the IR-S and IR-D groups (*p* < 0.05). The larger the epidermal layer length, the smaller the percentage of necrotic beak areas. In the process of IR-S and IR-D group beak sloughing, the epidermal layer gradually wraps the outer edge of the beak section (Figure 7), forming a barrier between the healed and necrotic tissues, avoiding the formation of open wounds after beak treatment and reducing the risk of infection. A similar healing processes was observed in turkeys that were IR-S beak treated (35). Laser beak trimming can remove the beak tip without an open wound, and the beak epidermis layer of the LAS group was able to completely wrap the whole beak within 1 week. These findings suggest that laser beak trimming enhances regrowth of the epithelialization. From 2 to 17 d of age, the number of herbal corpuscles in the CON group was significantly higher than in the other three experimental groups (*p* < 0.05). In the CON group, more Herbst corpuscles (*n* = 11–26) were visible at the tips of the bottom beaks, and this number also showed an increasing trend with the increasing age; further, there were fewer Herbst corpuscles (*n* = 0–4) than in the other three experimental groups. This finding indicated that the beak treatment damaged the beak neurons of chicks and that this damage was irreversible. These results are consistent with those of a study by Mckeegan et al. (27).

**Table 3:** Effect of different beak treatment on morphology of beak tissue in layer chick from 1 to 8wk.

Indictors	Age (Days)	Group				*P*
		IR-S	IR-D	LAS	CON	
Epidermis Thickness (μm)	2	49.8 ± 16.09	61.8 ± 13.30	54.70 ± 10.11	74.06 ± 11.28	0.324
Epidermis Thickness (μm)	7	59.29 ± 7.12	65.41 ± 12.61	71.70 ± 9.83	66.33 ± 17.91	0.417
Epidermis Thickness (μm)	17	67.0 ± 10.82^c^	98.0 ± 8.06^ab^	86.8 ± 7.99^b^	113.6 ± 10.46^a^	0.023
Percent of Epidermis length	2	26.1 ± 6.68^c^	35.9 ± 9.63^c^	72.8 ± 2.51^b^	100^a^	<0.001
Percent of Epidermis length	7	65.0 ± 7.71^b^	73.0 ± 11.32^b^	98.57 ± 6.85^a^	100^a^	0.004
Percent of Epidermis length	17	95.51 ± 5.23	100	100	100	0.291
Percent of necrotic beak tip area	2	44.7 ± 6.78^a^	33.0 ± 6.55^a^	0^b^	0^b^	0.006
Percent of necrotic beak tip area	7	8.1 ± 5.53^a^	6.3 ± 5.68^a^	0^b^	0^b^	0.004
Percent of necrotic beak tip area	17	0	4.7 ± 8.20	0	0	0.441
Number of Herbst corpuscles (pcs)	2	0.3 ± 0.58^b^	0^b^	0.7^b^	14.3 ± 5.51^a^	<0.001
Number of Herbst corpuscles (pcs)	7	0^b^	0^b^	1.5 ± 1.9^b^	10.8 ± 2.17^a^	<0.001
Number of Herbst corpuscles (pcs)	17	0.7 ± 1.15^b^	1.0 ± 1.00^b^	4.0 ± 2.65^b^	32.0 ± 8.89^a^	<0.001

IR-S, single-infrared energy source treatment; IR-D, double-infrared energy source treatment; LAS, laser energy source treatment; CON, sham untreated control. ^a,b,c^ Among 4 groups means within a main effect with different superscripts are significantly different (*P* < 0.05). All values are mean ± standard deviation.

### Growth performance and organ weight percentage

The effects of beak-treatment methods on the FI and BW of chicks are shown in Table 4. At 1 and 3 weeks of age, the FI of the CON group was significantly higher than that of the IR-S and IR-D groups, but at 6 weeks of age, the FI was significantly lower than that of the IR-S and IR-D groups (*p* < 0.05). Similarly, at 1–4 weeks of age, the BW of chicks in the CON group was significantly greater than that of the IR-S and IR-D groups (*p* < 0.05). However, at 6–8 weeks of age, no significant differences were observed for the BW of chicks among the CON group, IR-S and IR-D groups. Some previous studies have also reported that IR-S hens had lower BW and FI from 2 to 4 weeks of age (3, 10, 11). Similar results have been reported in a local broilers breed (36) and in Nicholas turkey hens (35). There were minimal significant differences in BW and FI between the CON and LAS groups from 1 to 8 weeks of age. Compared with the CON chicks, chicks that underwent the IR beak treatment showed a decreased FI, and often, there was a corresponding reduction in the BW (37, 38). However, LAS beak trimming showed no adverse effects on BW and FI in early-stage layer chicks. The BW at 1 week of age and the FI at 2 weeks of age in the IR-S group were significantly lower than those in the IR-D group. Conversely, at 3 and 6 weeks of age, the BW in the IR-S group was significantly higher than that of the IR-D group. No significant differences were observed in BW and FI between the IR-S and IR-D groups at other weeks of age. These findings suggest that IR beak treatments, both IR-S and IR-D, exert no lasting effects but do exhibit phased impacts on BW and FI.

**Table 4:** Effect of beak treatment on body weight and food intake in layer chicks from 1 to 8wk.

Indictors	Age (weeks)	Group				P
		IR-S	IR-D	LAS	CON	
BW (g)	1	51.11 ± 5.72^c^	53.20 ± 6.03^bc^	54.02 ± 4.89^ab^	56.06 ± 4.22^a^	0.005
	2	105.13 ± 5.31^c^	107.70 ± 4.28^b^	109.05 ± 3.50^ab^	110.47 ± 5.19^a^	<0.001
	3	167.30 ± 9.52^a^	165.04 ± 8.77^b^	166.59 ± 8.49^a^	169.74 ± 9.41^a^	0.074
	4	250.73 ± 13.21^b^	250.77 ± 13.32^b^	254.85 ± 13.37^ab^	258.19 ± 15.09^a^	0.528
	6	458.15 ± 23.92^a^	447.13 ± 18.89^b^	450.96 ± 19.42^ab^	451.26 ± 23.94^ab^	0.085
	8	657.46 ± 30.00	654.42 ± 32.60	656.50 ± 34.29	664.14 ± 37.89	0.510
FI (g)	1	7.96 ± 0.24^b^	8.19 ± 0.13^a^	8.19 ± 0.14^a^	8.47 ± 0.21^a^	0.018
	2	14.72 ± 0.24^b^	14.67 ± 0.23^b^	15.40 ± 0.47^a^	14.76 ± 0.20^b^	0.001
	3	19.14 ± 0.56^c^	19.56 ± 0.33^bc^	19.94 ± 0.64^ab^	20.22 ± 0.33^a^	0.006
	4	30.78 ± 1.00^b^	31.24 ± 1.13^b^	33.23 ± 1.61^a^	31.39 ± 0.87^b^	0.010
	6	41.30 ± 0.51^a^	40.76 ± 1.20^ab^	37.22 ± 1.34^c^	39.74 ± 1.28^b^	<0.001
	8	51.12 ± 1.98^ab^	50.17 ± 0.81^b^	52.38 ± 2.06^a^	51.97 ± 0.79^ab^	0.095

IR-S, single-infrared energy source treatment; IR-D, double-infrared energy source treatment; LAS, laser energy source treatment; CON, sham untreated control. BW, body weight; FI, feed intake. ^a,b,c^ Among 4 groups means within a main effect with different superscripts are significantly different (*P* < 0.05). All values are mean ± standard deviation.

**Figure 8:**
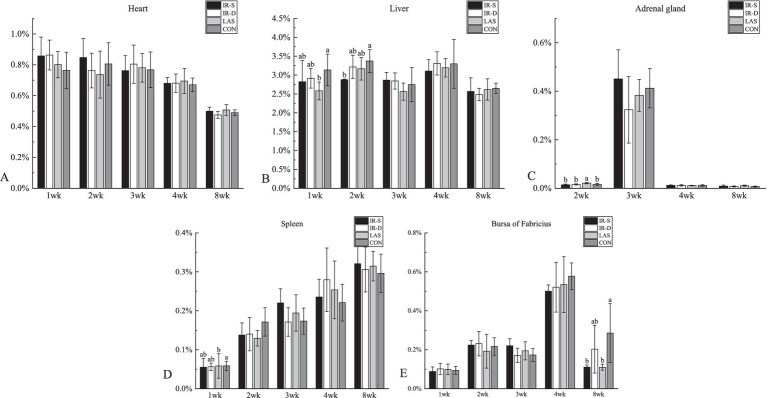
Effect of different beak treatment on the organ weight percentage of chick from 1 to 8 wk. IR-S: single-infrared energy source treatment, IR-D: double-infrared energy source treatment, LAS: laser energy source treatment, CON: sham untreated control. **(A–E)** Column chart of heart, liver, adrenal gland, spleen, bursa of fabricius weight percentages. ^a,b^ Among 4 groups mean organ weight percentages at the age of 1–8 weeks layer chicks without a common superscript differ (*P* < 0.05).

The effects of different beak-treatment methods on the chick organ weight percentages are shown in Figure 8. The liver percentage at 1 week in the CON group was significantly higher than in the LAS group, the liver weight percentage (Figure 8B) at 2 weeks in the CON group was significantly higher than that in the IR-S group, and bursa of Fabricius percentage (Figure 8E) at 8 weeks of age was significantly higher than that in the IR-S and LAS groups (*p* < 0.05). Studies by Onbasilar et al. (39) and Li et al. (36) have demonstrated that the liver and spleen weights are affected by beak-treatment methods. However, the weight percentage of the bursa, an immune organ, decreased in the experimental groups at 8 weeks of age; this may be associated with the differential effects of beak treatment on various immune organs. Furthermore, the scarcity of published data on the impact of IR-S beak treatment on organ weight percentages made further comparisons difficult.

## Conclusion

Compared with the CON group, beak treatment procedures resulted in a reduction of beak length from 1 to 8 weeks of age, enhanced beak symmetry, diminished the quantity of Herbst corpuscles, and decreased the organ weight percentages of the liver and bursa of Fabriscus. IR-S and IR-D beak treatment decreased the early BW and FI in layer chicks. Compared with IR-S and IR-D beak treatment, LAS beak treatment results in faster beak growth and better beak symmetry. Compared with the CON group, LAS beak treatment did not affect the BW and FI of layer chicks from 1 to 8 weeks of age. Compared with IR-D beak treatment, the peak time of bottom beak sloughing in the IR-S group was earlier and longer. Simultaneously, there were differences in beak length, BW, and epidermal thickness from 2 to 3 weeks of age between the LAS and CON groups, but there were no differences in other indicators.

## Data Availability

The original contributions presented in the study are included in the article/supplementary material, further inquiries can be directed to the corresponding author.
